# Clonal Hematopoiesis Is Associated With Cardiomyopathy During Solid Tumor Therapy

**DOI:** 10.1016/j.jaccao.2024.05.013

**Published:** 2024-07-02

**Authors:** Etienne Leveille, Rachel Jaber Cheheyeb, Carlos Matute-Martinez, Nathan W. Chen, Ritujith Jayakrishnan, Anthos Christofides, Derrick Lin, Yunju Im, Giulia Biancon, Jennifer VanOudenhove, Stephanie Halene, Jennifer M. Kwan

**Affiliations:** Yale University School of Medicine, New Haven, Connecticut, USA

Clonal hematopoiesis of indeterminate potential (CHIP) is characterized by the presence of a clonal, nonmalignant population of myeloid cells. These cells contribute to an inflammatory state, leading to an increased rate of adverse cardiovascular events in individuals with CHIP.^1^, ^2^, ^3^ However, it is unclear how CHIP affects the cardiovascular outcomes of patients with solid cancers.^4^ Thus, we sought to characterize the associations between CHIP and prevalent cardiovascular risk factors and disease and incidence adverse cardiovascular events during cancer therapy.

In a prospective study approved by the Yale Institutional Review Board, patients undergoing treatment for solid tumors were enrolled after informed consent was obtained. Patients with active, blood-borne cancers were excluded. CHIP status at the time of enrollment was assessed through blood whole exome sequencing using a dedicated gene panel of known CHIP genes,^2^^,^^5^ with a median of 1,000× sequencing depth coverage. The primary cardiovascular outcome evaluated was the development of cardiomyopathy, defined as new ejection fraction <50% on echocardiography or cardiac magnetic resonance imaging. For patients with underlying cardiomyopathy, a left ventricular ejection fraction decline >10% from baseline met the criteria as new cardiomyopathy. Descriptive statistics were used to compare baseline characteristics in CHIP vs no CHIP. To define the associations between CHIP status and primary cardiovascular outcomes, Kaplan-Meier survival analyses and multivariable competing risk Cox regression modeling were performed to determine subdistribution HRs with 95% CIs. Covariates included age; sex; prevalent history of hyperlipidemia; hypertension; obstructive coronary artery disease (CAD); diabetes; metastatic disease; chest radiation therapy; and the use of an immune checkpoint inhibitor (ICI), anthracycline, or human epidermal growth factor–positive therapies.

Overall, 236 patients, all of whom had an inpatient or outpatient cardio-oncology consultation, were enrolled. Of these, 51 (21.6%) were enrolled before cancer therapy. A total of 90 patients (38.0%) harbored CHIP, which was higher than expected in comparison to a general population of similar age but similar to previous series of cancer patients.^1^^,^^4^ *DNMT3A* and *TET2* were the most frequently mutated genes; our cohort showed a lower rate of *ASXL1* sequence variants and a higher rate of *IDH2* sequence variants compared to prior series.^1^ The average age was 63.7 ± 13.3 years among the entire cohort, and CHIP patients were older (CHIP: 67.6 ± 13.5 years, no CHIP: 61.3 ± 12.6 years; *P* < 0.001). There was no difference in sex between CHIP or non-CHIP patients; 71.2% of subjects were female (CHIP: 64.4%, no CHIP: 75.3%; *P =* 0.078).

The most common cancer types were breast at 41.9% (CHIP: 32.2%, no CHIP: 47.9%; *P =* 0.021), genitourinary at 14.4% (CHIP: 22.2%, no CHIP: 9.6%; *P =* 0.012), lung at 15.8% (CHIP: 15.6%, no CHIP: 15.8%; *P* > 0.99), and melanoma at 5.9% (CHIP: 7.8%, no CHIP: 4.8%; *P =* 0.40). Although not statistically significant, the burden of metastatic disease (35.2%) was higher in the CHIP group (CHIP: 41.1%, no CHIP: 31.5%; *P =* 0.16). The use of potentially cardiotoxic therapies included human epidermal growth factor 2–targeting agents in 15.3% (CHIP: 12.2%, no CHIP: 17.1%; *P =* 0.36), anthracyclines in 27.5% (CHIP: 30.0%, no CHIP: 26.0%; *P =* 0.55), ICI in 37.3% (CHIP: 46.7%, no CHIP: 31.5%; *P =* 0.026), and chest radiation therapy in 48.7% (CHIP: 42.2%, no CHIP: 52.7%; *P =* 0.14) of patients.

At baseline, obstructive CAD had a similar prevalence in both groups (CHIP: 11.1%, no CHIP: 10.9%; *P* > 0.99). The rate of heart failure with preserved ejection fraction was significantly higher in the CHIP group (CHIP: 20.0%, no CHIP: 7.5%; *P =* 0.007).There were no other significant differences in other cardiac diseases or risk factors, including heart failure with reduced ejection fraction (CHIP: 17.8%, no CHIP: 13.0%; *P =* 0.35), diabetes (16.5%; CHIP: 16.7%, no CHIP: 16.4%; *P* > 0.99), hypertension (54.7%; CHIP: 56.7%, no CHIP: 53.4%; *P =* 0.69), and hyperlipidemia (47.0%; CHIP: 51.1%, no CHIP: 44.5%; *P =* 0.35). In terms of imaging, 216 patients (91.5%) had a baseline transthoracic echocardiogram or cardiac magnetic resonance imaging performed, and 177 (75.0%) had follow-up imaging study.

The last follow-up date was December 1, 2023, with a median follow-up of 570 days (Q1-Q3: 297-863 days) and a maximum of 2,009 days. New cardiomyopathy occurred in 16.5% of patients and was more frequent in patients with CHIP (CHIP = 35.6%, no CHIP = 4.8%, crude rates). The cumulative incidence of cardiomyopathy was greater in patients with CHIP (CHIP = 38% [Q1-Q3: 24%-53%], no CHIP = 25% [Q1-Q3: 6.3%-50%]; *P* < 0.001 by Gray's test). The median time to cardiomyopathy was 441 days (Q1-Q3: 158-800 days) in the CHIP group compared to 614 days (Q1-Q3: 388-868 days) in those without CHIP (*P <* 0.001). Additionally, cumulative incidence curves revealed a significant difference in cardiomyopathy incidence between patients with and without CHIP by Gray's test (Figure 1A). In competing risk Cox regression models, CHIP was an independent predictor for the development of cardiomyopathy (HR: 2.01; 95% CI: 1.03-3.93; *P =* 0.042) after adjusting for CAD, cardiovascular risk factors, and oncologic therapies (Figure 1B). ICI use was also an independent predictor (HR: 1.95; 95% CI: 1.03-3.71; *P =* 0.042).

**Figure 1:**
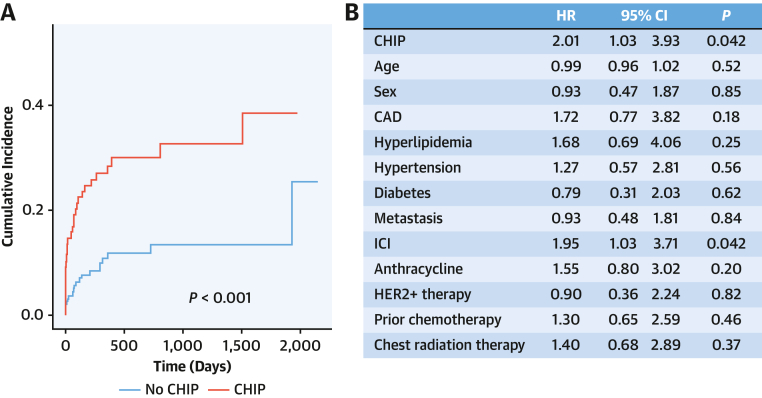
CHIP Is Associated With the Development of Cardiomyopathy During Cancer Treatment (A) Cumulative incidence curves showing the development of cardiomyopathy by clonal hematopoiesis of indeterminate potential (CHIP) status. *P* < 0.001 by Gray's test for the association between CHIP and cumulative incidence of cardiomypathy. (B) Multivariable competing risk Cox regression model for cardiomyopathy. CAD = coronary artery disease; HER2 = human epidermal growth factor 2; ICI = immune checkpoint inhibitor.

In summary, in this single-center, solid tumor cohort with a high prevalence of breast cancer, CHIP was a risk factor for the development of cardiomyopathy during cancer therapy. Cardiovascular events remain a major cause of morbidity and mortality in cancer patients, and the role of CHIP in the cardiovascular management of cancer patients remains an important, clinically relevant question.^3^^,^^4^ Our findings call for larger studies to define the role of CHIP in risk prediction, stratification, and management of cancer patients to optimize clinical outcomes and reduce treatment-related complications.

## Funding Support and Author Disclosures

This study was supported by the National Center for Advancing Translational Science (Clinical and Translational Science Awards grant UL1 TR001863) a component of the National Institutes of Health to Dr Kwan. The authors have reported that they have no relationships relevant to the contents of this paper to disclose.
